# Genetic Variants Associated with Neuropeptide Y Autoantibody Levels in Newly Diagnosed Individuals with Type 1 Diabetes

**DOI:** 10.3390/genes13050869

**Published:** 2022-05-12

**Authors:** Sara Juul Mansachs, Sofie Olund Villumsen, Jesper Johannesen, Alexander Lind, Simranjeet Kaur, Flemming Pociot

**Affiliations:** 1 Department of Children Adolescents, Copenhagen University Hospital Herlev, 2730 Herlev, Denmark; sara.juul.mansachs@regionh.dk (S.J.M.); jesper.johannesen@regionh.dk (J.J.); 2 Department of Clinical Medicine, Faculty of Health and Medical Sciences, University of Copenhagen, 1165 Copenhagen, Denmark; 3 Translational Type 1 Diabetes Research, Steno Diabetes Center Copenhagen, 2739 Herlev, Denmark; sofie.olund.villumsen.02@regionh.dk (S.O.V.); simranjeet.kaur@regionh.dk (S.K.); 4 Department of Clinical Sciences, Lund University CRC, Skåne University Hospital, 200 49 Malmo, Sweden; alexander.lind@med.lu.se; 5 EU Interreg, Medicon Valley Alliance, 2300 Copenhagen, Denmark

**Keywords:** NPY-L autoantibodies, minor autoantigen, autoimmunity, type 1 diabetes, genetic regulatory mechanisms, GWAS

## Abstract

**(1)** Autoantibodies to the leucine variant of neuropeptide Y (NPY-LA) have been found in individuals with type 1 diabetes (T1D). We investigated the association between the levels of NPY-LA and single nucleotide polymorphisms (SNP) to better understand the genetic regulatory mechanisms of autoimmunity in T1D and the functional impacts of increased NPY-LA levels. **(2)** NPY-LA measurements from serum and SNP genotyping were done on 560 newly diagnosed individuals with T1D. SNP imputation with the 1000 Genomes reference panel was followed by an association analysis between the SNPs and measured NPY-LA levels. Additionally, functional enrichment and pathway analyses were done. **(3)** Three loci (*DGKH*, *DCAF5*, and *LINC02261*) were associated with NPY-LA levels (*p*-value < 1.5 × 10^−6^), which indicates an association with neurologic and vascular disorders. SNPs associated with variations in expression levels were found in six genes (including *DCAF5*). The pathway analysis showed that NPY-LA was associated with changes in gene transcription, protein modification, immunological functions, and the MAPK pathway. **(4)** Conclusively, we found NPY-LA to be significantly associated with three loci (*DGKH*, *DCAF5*, and *LINC02261*), and based on our findings we hypothesize that the presence of NPY-LA is associated with the regulation of the immune system and possibly neurologic and vascular disorders.

## 1. Introduction

Type 1 diabetes (T1D) is an autoimmune disease caused by the destruction of pancreatic islet β cells [1]. The immune-mediated attack on β cells is caused by complex interactions between environmental factors and multiple risk-conferring genes [2]. More than 75 susceptibility loci for T1D have been identified with genome-wide significance [3]. The human leukocyte antigen (HLA) region on chromosome 6p21 contributes to approximately 50% of the familial genetic risk [4], and the HLA region confers the strongest risk for islet autoantibodies which are biomarkers of T1D autoimmunity [2,5,6]. *HLA-DR3-DQ2* is associated with autoantibodies to glutamic acid decarboxylase (GADA) as the first appearing islet autoantibody [7]. Additionally, the presence of autoantibodies to islet antigen-2 (IA-2A) is associated with *HLA-DR4-DQ8* [2,5,6,8] but is negatively associated with *HLA-DQ2* [9].

Other loci, identified by genome-wide association studies (GWAS), have minor individual effects on the total genetic risk for T1D, but for most, their biological functions are still unknown [7]. Fine-mapping has identified T1D credible variants to be strongly enriched and affecting expression in T cells [3] and T cell activated B cells which produce islet autoantibodies [6]. Genes such as *IFIH1* [10], *ERBB3*, *SH2B3*, *RBFOX1*, *BACH2* [3,5], *PTPN22*, *CTLA4*, *CCR7*, *TNFAIP3*, and *CD226* [7,11], are associated with GADA as the first appearing islet autoantibody [10], most of them in *HLA-DR3-DQ2/HLA-DR3-DQ2* individuals [11]. In addition, 3q28/*LPP* is associated with persistent GADA [12].

Individuals with IA-2A are at increased risk for T1D progression [13], and the IA-2A-associated genes 1q23/*FCRL3* and 11q13/*RELA* are assumed as candidates for early screening of clinical onset of T1D [12,14], while *IFIH1* is negatively associated with IA-2A [10].

The known T1D autoantibodies represent only one part of the multifaceted autoimmune response towards β cells [15]. There are still many unknown mechanisms indicating that T1D is a much more diverse disease with several endotypes [2]. In unraveling the disease complexity, autoantibodies to minor T1D autoantigens have been investigated [15,16,17].

Neuropeptide Y (NPY) is a vesicle cargo peptide [18], and antibodies against this autoantigen are found in 8–21% of individuals with T1D, but it is still unknown which role it plays in T1D autoimmunity [15,19]. It is an orexigenic endogenous 36 amino acid peptide found in neurons [20] and β-cell secretory granules (SGs) [21]. Expression of NPY is high in immature β cells but downregulated upon β-cell maturation to reemerge after T1D onset [21]. NPY inhibits glucose-stimulated insulin secretion and promotes β-cell replication through extracellular signal-regulated kinase activation [20,21,22,23,24]. Furthermore, it decreases cAMP in the sympathetic nervous system, thus, controlling the function of many organs [25]. NPY is found in two variants caused by a single nucleotide polymorphism (SNP) at the peptide signal region of NPY (rs16139, T1128C), where leucine (NPY-L) is substituted with proline (NPY-P) [19]. Of individuals with T1D, 90% carry the genotype coding for NPY-L [19].

Autoantibodies to NPY-L (NPY-LA) are present in both individuals with T1D and type 2 diabetes (T2D) [22] but also in healthy siblings to individuals with T1D [26], thus, suggesting NPY-LA has a genetic component [26]. Moreover, NPY-LA is positively associated with GADA and in children with T1D with increasing HbA1c levels over time (unpublished data) [26]. Interestingly, higher NPY autoantibodies have been found in *HLA-DQ8* positive individuals with T1D who are diagnosed over 10 years of age, while associations between NPY-LA and *HLA-DRB* or *-DP* have not yet been investigated [19].

In the present study, we aimed to characterize associations between NPY-LA levels and GWAS data, including their functional annotation. This was done to better understand the genetic regulatory mechanisms of autoimmunity and the functional impacts of having increased levels of the minor autoantibody, NPY-LA.

## 2. Materials and Methods

**Participants:** A total of 569 newly diagnosed individuals with T1D were available from three sub-cohorts, all part of The Danish Childhood Diabetes Register (DanDiabKids), described elsewhere [26,27,28]. Disease onset was defined as the date of the first insulin injection, and samples were taken within three months after the clinical onset of T1D.

The first sub-cohort, Steno 2007, consisted of 500 individuals with T1D, of which 77 were excluded due to insufficient material. The second sub-cohort included 129 consecutively enrolled individuals with T1D from the Danish Remission Phase Study, where 58 individuals were excluded due to insufficient material, co-participation in Steno 2007, or missing data. Both the first and second sub-cohorts had previously been genotyped with the ImmunoChip array. The third sub-cohort included 79 individuals with T1D from the CPH remission phase 2015 cohort [29], previously genotyped with GWAS, and four individuals were excluded due to insufficient material. Furthermore, nine individuals with T1D were excluded due to missing information from GWAS- or ImmunoChip-analyses.

Participant demographics of all the 560 individuals with T1D are shown in Table 1. This study, including the existing protocols from DanDiabKids, Steno 2007, the CPH remission phase 2015 cohort, and the Danish Remission Phase Study, has been carried out per the Declaration of Helsinki, and all were approved by the Danish Ethical Committee and by The Danish Data Protection Agency.

**Autoantibody measurements:** Serum samples from the 560 individuals, taken within 3 months from clinical onset of T1D, have been stored at −80 °C and were analyzed for NPY-LA using a radioligand binding assay (RBA), described elsewhere [22,26,30]. In brief, NPY-L was subjected to coupled in vitro transcription-translation to generate ^35^S-methionine radiolabeled autoantigens incorporated into an overnight immunoprecipitation/filtration assay. A polyclonal rabbit IgG antibody to NPY (Abcam, Cambridge Science Park, Cambridge, UK), in seven-doubling dilution steps, was used for converting Protein A Sepharose-bound radioactivity into in-house units. Analyses for NPY-LA were done in duplicates. The mean value of the intra-assay coefficient of variation (CV) for duplicates was 13%. The NPY-LA assay has not been part of the international standardization workshops [31,32].

GADA and IA-2A were analyzed within 3 months from the clinical onset of T1D and were analyzed in a standard RBA, previously described in detail elsewhere [28,32]. Levels were used instead of dichotomous values since GADA quartiles above Q1 show increasing NPY-LA values [26]. Additionally, individuals with IA-2A in the upper quartile compared to the lower quartile have a fourfold risk of developing T1D [6]. Titration above 500 U/mL for GADA and 250 U/mL for IA-2A have not been performed [28]. However, undiluted values above the standard curves were used [6,31,33]. Levels above the standard curve are underestimated as end-point titrations tend to attain higher levels.

**SNP genotyping of the three sub-cohorts:** A total of 560 individuals had genotyping data available, of which 486 were previously genotyped on the ImmunoChip array described elsewhere [12], and 74 were genotyped on the Illumina Infinium Global Screening Array (GSA version 24.v2.0). Before quality control (QC), the ImmunoChip array dataset and GSA array dataset had 177,022 and 730,059 SNPs, respectively. After QC and frequency pruning (–geno 0.1 –maf 0.05 –hwe 0.001 –mind 0.05) using PLINK v1.90, the ImmunoChip and GSA datasets had a genotyping rate of 0.99 and had 109,021 SNPs and 306,455 SNPs, respectively.

The two datasets were merged using PLINK v1.90 before proceeding with the imputation. A total of 13,066 SNP markers overlapped between the two platforms. Before merging, we made sure that both datasets had the same strand and build concordance. After QC and strand alignment, 402,410 SNPs remained in the merged dataset. After removing duplicate variants, 397,929 SNPs remained for imputation analysis.

**Imputation with 1000 genomes:** To explore regions not well covered by the ImmunoChip, imputation was performed on the merged dataset using the 1000 Genomes haplotype reference panel. Chromosome strand and allele order in the merged dataset were adjusted to match the reference 1000 Genome Phase 3 dataset (excluding non-European samples) using the conform-gt program. Pre-phasing was performed using SHAPEIT v2 [34] (with European population (–effective-size = 11,418)). Imputation was then performed using IMPUTE2 v2.3.2 [35] and 1000 Genome phase3 panel [36] with default parameters. For imputation to run efficiently, each chromosome was split into 5 MB chunks for analysis, as the IMPUTE2 program produces higher accuracy over short genomic regions. The IMPUTE2 results were obtained in a gene/sample output format. The chromosome chunks were concatenated for each chromosome and converted to VCF format using BCFtools v1.8. The annotation of the VCF file was done with the annotate function in BCFtools using the dbsnp version150 (build GRCh37p13). PLINK was used to filter insertion-deletion mutations (INDELS), all variants with 2+ alternate alleles, and SNPs with no-call alleles. The files were then converted to PLINK bed format. To assess imputation quality, the IMPUTE2 internal quality metrics (INFO score) were obtained. The INFO values range from 0 to 1, where a higher value indicates the increased quality of an imputed SNP. A total of 3,119,616 SNPs remained after post imputation QC and after filtering low imputation score SNPs (INFO < 0.4).

**Association and pathway analysis:** Linear regression was used to evaluate the association between the imputed genetic variants and NPY-LA. Out of the 560 samples, 11 Non-European samples were removed before proceeding with the association analysis. A quantitative association test, using a linear function in PLINK v 1.90, was performed on the NPY-LA measurements in 546 out of 549 samples (3 samples had missing values) and the imputed SNPs (*n* = 3,119,616). The linear model was adjusted for the following covariates: sex, age at clinical onset of T1D, ethnicity, log2 of GADA, and IA-2A measurements. Ethnicity was coded as a factor with two levels (1 = Danish, 2 = European/Turkish). The age at clinical onset of T1D was grouped into four levels: below 5, 5–10 or 10–15 years, and above 15 years. The value of genomic inflation-estimate (lambda), a measure of inflated *p*-values (based on median chisq), was 1. Lambda statistic should be close to 1 if the points fall within the expected range or greater than 1 if the observed *p*-values are more significant than expected. Manhattan plot and QQ-plots before and after adjustment for genomic control were created using the qqman package in R version 4.1.0. A *p*-value cutoff of *p* < 1.0 × 10^−4^ was used to identify the top signals from the association analysis.

Variant Effect Predictor (VEP) in Genome Reference Consortium Human Build 38 Ensembl release 104 (GRCh38) was used to annotate the top variants. Expression quantitative trait loci (eQTLs) in whole blood were extracted for the top variants using the GTEx portal (The Genotype-Tissue Expression Project), hereby retrieving *p*-values and normalized effect sizes (NES). The NES of eQTLs is defined as the slope of the linear regression. It is computed as the effect of the alternative allele relative to the reference allele in the human genome reference GRCh38/hg38. A *p*-value cutoff of *p* < 0.05 was used as the threshold for the eQTL analysis.

Pathway analysis and network establishment were performed on the annotated top variants using Ingenuity Pathway Analysis (QIAGEN IPA, RRID: SCR_008653).

## 3. Results

Of the 560 individuals with T1D, 0.5% had missing NPY-LA values (3/560). For GADA, 53% and 21% were positive and negative, respectively. IA-2A was detected in 51% of individuals, while 23% were negative. The measurements were missing in 26% for both GADA and IA-2A, and the missing values of the two variables were set to zero. The ethnic distribution of the genotyped samples is shown in Table S1.

**Figure 1:**
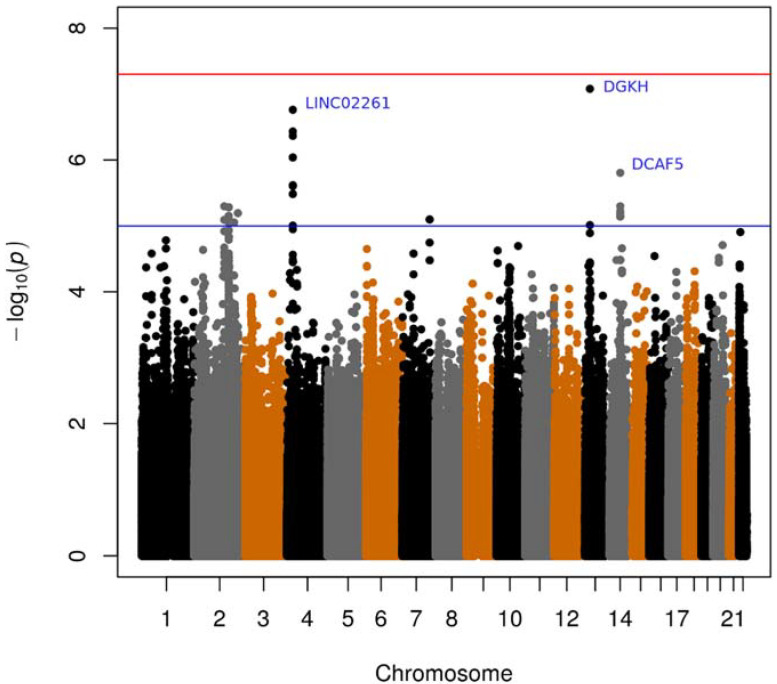
Manhattan plot of the genome-wide association study (GWAS). Manhattan plot illustrating the associations of NPY-LA with all the SNPs from the three sub-cohorts. *Abbreviations:* NPY-LA, Autoantibodies to neuropeptide Y; SNPs, single nucleotide polymorphisms. Blue line: suggestive line (*p*-value < −log_10_(1 × 10^−5^)); red line: genome-wide line (*p*-value < −log_10_(5 × 10^−8^)). The black, grey and orange dots represents SNPs, and these three colors alternate for the chromosomes.

The association analysis between the NPY-LA measurements and the genotyped score of each SNP is illustrated in a QQ-plot (Figure S1) that demonstrates observed and expected −log10 *p*-values. A Manhattan plot illustrates chromosomes with significant SNPs (Figure 1).

Several SNPs mapped to the intergenic region on chromosome 4 upstream of *LINC02261* (Table 2). Another signal of significance for NPY-LA was the SNP (rs2148655) on chromosome 13 mapping to *DGKH* (Table 2). Furthermore, the SNP (rs1275406) on chromosome 14 mapped to *DCAF5* (Table 2). None of the three genes have previously been associated with T1D.

The annotation of the top SNPs (*p* ≤ 1.01 × 10^−4^) resulted in 348 SNPs (Table S2), mapping to 52 genes. These variants mapped to intron sequences (51%), intergenic sequences (2%), regulatory regions (2%), non-coding transcripts (30%), upstream gene variants (6%), downstream gene variants (5%), non-coding transcript exon variants (1%), and NMD transcript variant (3%).

Expression quantitative trait loci (eQTLs) for the selected 348 SNPs in whole blood were retrieved from GTEx, and the analysis identified several eQTLs of *DCAF5* (Table 3). Only 24 out of the 348 selected variants had eQTL effects on the host gene, and the majority of these (19 variants) mapped to the *DCAF5* locus. Other loci with eQTL variants included *ABCC2*, *CCDC6*, *PABPC1L*, *DENND2C*, and *TTF2* (Table 3, *p*-value < 0.05).

We identified a set of top molecules based on pathway analysis of the 348 SNPs (using IPA), mapping to 52 genes (Table S3). We highlight the presence of SNP rs79095830 from host gene *ABCC2* and SNP rs3746583 from gene *DCAF5,* of which eQTL was identified (Table 3 and Table S3).

**Figure 2:**
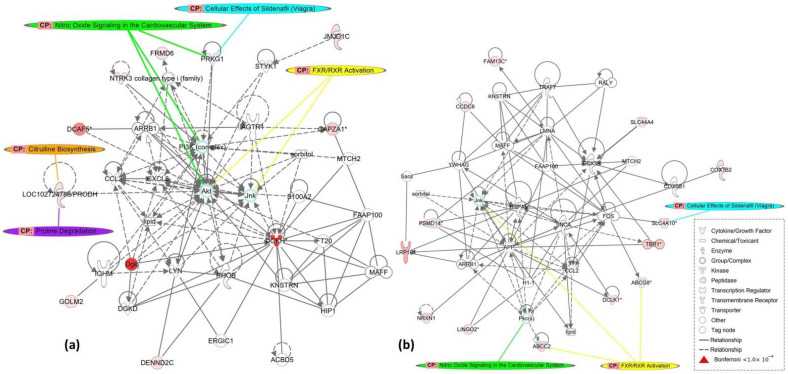
Networks from Ingenuity Pathway Analysis (IPA). (**a**) Network which includes *DCAF5*, *DGKH*, *LYN,* and the MAPK pathway. (**b**) Network which includes *PSMD14* and JNK. *Abbreviation*: *DGKH*, diacylglycerol kinase enzyme family; *DCAF5*, DDB1, and CUL4 associated factor 5; MAPK, p38 mitogen-activated protein kinases; PI3K, phosphoinositide 3-kinase. *Note:* Arrows denote activation/expression. The canonical pathways (CP) illustrated in the networks correspond to the top CPs that the significant SNPs mapped to.

Finally, the IPA analysis generated two networks that illustrate the influence of the NPY-LA associated genes on the p38 mitogen-activated protein kinases (MAPK) pathway, where one network includes JNK, Akt, and PI3K (Figure 2a) and the other includes JNK (Figure 2b).

## 4. Discussion

This observational study of 569 children with T1D was designed to examine genetic loci associated with levels of NPY-L autoantibodies at the clinical onset of T1D. Association analysis identified three loci *DGKH*, *DCAF5*, and *LINC02261*, which had SNPs significantly associated with NPY-LA levels. When including GADA and/or IA-2A levels in the model, the associations did not change, indicating that the three loci are not important for the known association between NPY-LA and the islet autoantibodies [26]. The functional enrichment analysis (eQTL) identified SNPs associated with variations in expression levels in whole blood of *DCAF5*, *ABCC2*, *CCDC6*, *PABPC1L*, *DENND2C*, and *TTF2,* suggesting potential interactions between these genetic factors and NPY-LA levels. The top molecules of the pathway analysis included *DGKH*, *DCAF5*, *PABPC1L*, and *ABCC2*.

The gene, *DGKH*, encodes a member of the diacylglycerol kinase enzyme family [37], while *DCAF5* encodes DDB1 and CUL4-associated factor 5 [38], and *LINC02261* is a non-protein-coding lncRNA. None of the genes are known susceptibility loci for T1D or islet autoimmunity. However, *DCAF5* has previously been linked with T2D [39], and NPY-LA has been found in individuals with T2D [22], suggesting NPY-LA influences diabetic processes shared between T1D and T2D. *ABCC2* is part of the adenosine triphosphate-binding cassette family (ABC transporters) located in the plasma membrane that enables an active transport of various compounds across the cell membrane [40,41]. Both the pathway top molecules and the pathway networks indicate that NPY-LA is associated with changes in the mitogen-activated protein kinase (MAPK)-signaling pathway, gene transcription, protein modification, and immunological functions.

**MAPK-signaling pathway:** Several identified genes affect cell transcription and apoptosis signaling. *DGKH* is known to regulate intracellular concentrations of phosphatidic acid and diacylglycerol and has a key role in promoting cell growth by activating the MAPK-signaling pathway [37]. MAPKs are implicated in altered transcriptions of genes and, thus, numerous cellular behaviors such as survival, proliferation, differentiation, and apoptosis [42,43]. The network identifies *DGKH* as a hub node directly or indirectly activating several kinase signaling pathways, e.g., Akt, JNK, PI3K, and LYN. Akt affects glucose uptake and utilization, glycogen, fatty acid, and protein syntheses, and has been implicated in several diseases, including diabetes, autoimmunity, and insulin resistance [44,45]. The c-Jun N-terminal kinase (JNK) pathway is one of the major signaling cassettes of the MAPK signaling pathway and plays an important role in apoptosis, inflammation, cytokine production, and metabolism [46]. The Phosphoinositide 3-kinase (PI3K) complex is linked to an extraordinarily diverse group of cellular functions but has an important role in glucose regulation and may be involved in the clinical onset of T1D [47]. *LYN* is a member of the Src family of tyrosine kinases and plays an important role in regulating innate and adaptive immune responses, responses to growth factors, and cytokines [48]. Interestingly, *LYN* was recently identified as a novel T1D susceptibility locus [3]. Also, *DCAF5* indirectly interacts with Akt. Other genes from the pathway analysis and eQTLs support an association with the MAPK-signaling pathway (*CCDC6*, *PRKG1*, *TTF2*, *PABPC1L*, *SND1*, and *ABCC2*). This suggests that NPY may impact the T1D phenotype by complex regulation of pro- and anti-apoptotic signaling pathways.

Furthermore, the MAPK pathway is regulated by enzymatic post-translational modification of ubiquitination [49,50], a proteasomal degradation to regulate the activity of targeted proteins [41]. *DCAF5* is assumed to function as a substrate receptor for the CUL4-DDB1 E3 ubiquitin-protein ligase complex helping ubiquitination [38]. Ubiquitin chains have been shown to play important roles in inflammatory signaling pathways [51], and interestingly, both *DGKH* [38] and *DCAF5* influence neutrophiles of the innate immune system. Studies have shown that increasing NPY levels in mice lead to hyperglycemia, impaired glucose tolerance, hyperphagia, and obesity. On the contrary, NPY knockdown in rats leads to the development of brown adipose tissue and elevated thermogenic activity [21,52,53,54,55]. Additionally, injection of NPY autoantibodies in mice decreases food consumption [56].

The findings suggest that NPY-LA plays a role in post-translational modification and regulation of the MAPK signaling pathway via ubiquitination, and through that influence survival of β cells or activation of inflammatory cells, thus influencing the autoimmune response in T1D and glucose regulation. Interestingly, NPY acts through the Y1 receptor to increase neuronal proliferation, mediated through activation of the MAPK pathway [57].

**Neurological disorders:** *DCAF5* has been associated with neurogenic bowel disease [58], which is interesting since 20% of individuals with T1D in time will experience neurogenic bowel disease due to irreversible autonomic neuropathy [59]. Furthermore, prior studies have shown that defects in the expression level or stability of ABC transporters result in neuropathies [41]. Moreover, individuals with long-duration T1D are complicated with autonomic neuropathy when they have markedly reduced or absent responses of NPY to insulin-induced hypoglycemia [60]. Since we assume that NPY levels are diminished in the presence of NPY-LA [61], we hypothesize there is a possible association between NPY-LA levels, neuropathy, gene variants of *DCAF5*, and the function of *ABCC2*. Although, a previous study including few participants with T1D and T2D found no relation between peripheral or cardiac autonomic neuropathy and NPY autoantibodies [22]. Studies of NPY-LA with more participants having long-duration T1D and T2D and including identifications of genetic variants are needed to further elaborate associations between *DCAF5*, *ABCC2*, NPY-LA, and neuropathies.

The present study identified a significant association between NPY-LA and *DGKH*, a gene that has previously been reported in bipolar disorders [62]. Also, the lncRNA *LINC02261* has previously been found in schizophrenia and autism spectrum disorder [63,64]. Interestingly, several genes that were previously found to affect the MAPK-signaling pathway are known to be associated with schizophrenia, schizoaffective, and bipolar disorders [65]. Moreover, prior studies have shown that numerous lncRNAs and their target genes modulate signaling pathways known to play a role in T1D-related neuronal dysfunctions [66].

Additionally, our pathway analysis identified genes affecting calcium channels (*PRKG1* [67]). NPY acts to modulate voltage-gated Ca^2+^-channels in the neuronal system [68], and, interestingly, inhibition of glucose-stimulated insulin secretion by NPY is assumed to be dependent on Ca^2+^ in β cells [24]. Voltage-gated calcium channels are present in the membrane of neurons, β cells, and muscle cells (skeletal, cardiac, and smooth). They regulate intracellular processes such as neurotransmission, secretion, and contraction. Besides, Ca^2+^ is involved in the vesicular trafficking of ABC transporters to the membrane [41].

In T1D, persistent hyperglycemia has previously been found to promote neurovascular dysfunction, leading to endothelial cell dysfunction, oxidative stress, increased neuronal cell apoptosis, and inflammation [59,66]. The association with endothelial dysfunction is supported by the MAPK-signaling pathway, an important regulator of inflammation on endothelial cells and the progression of atherosclerosis [69]. Interestingly, both mutations in the ABC transporter and the minor allele of NPY (NPY-P) have been associated with atherosclerosis [70,71]. Future genetic analyses of NPY-LA associations in individuals with T2D, who have neuropathy and vascular disorders, may elaborate on possible associations.

The strength of the present study involves the large number of samples at the clinical onset of T1D with genetic information to identify loci associated with NPY-LA levels in individuals with T1D. Though the lack of replication sample/data is a limitation, as well as the use of whole blood for functional annotation in the absence of follow-up experiments in relevant cells/models, an important strength is the combination of analyses identifying associations with SNPs, expressions, and pathway analyses. Finally, there may be limitations to using publicly available databases as study design and demographic data vary.

## 5. Conclusions

In conclusion, NPY-LA is significantly associated with three loci: *DGKH*, *DCAF5*, and *LINC02261*. It is hypothesized that the presence of NPY-LA is associated with activation of the innate immune system, where NPY-LA may interact with the MAPK pathway in the regulation of glucose homeostasis, inflammation, cell survival, ABC transporter, and Ca^2+^ levels in β cells, leukocytes, endothelial cells, and neurons in the development of T1D and possibly in T2D neuropathies or vascular disorders.

## Figures and Tables

**Table 1:** Participant demographics. *Abbreviations*: IA-2A, tyrosine phosphatase autoantibodies; GADA, glutamic acid decarboxylase autoantibodies.

Variables	*n*	Percent	Mean
**Age**	560		10 years (range 0–18 years)
**Sex**			
Male	301	54%	
Female	259	46%	
**Ethnicity**			
Scandinavian	536	96%	
European or Turkish	13	2%	
Other	11	2%	
**GADA**			
Positive	299	53%	
Negative	115	21%	
Missing	146	26%	
**IA-2A**			
Positive	288	51%	
Negative	126	23%	
Missing	146	26%	

**Table 2:** Top 10 of NPY-LA associated SNPs in 546 children with T1D at clinical onset (1–3 months disease duration). *Abbreviation*: T1D, type 1 diabetes; NPY-LA, Autoantibodies to neuropeptide Y;; SNP, Single nucleotide polymorphism; ADD, additive effects of allele dosage; BP, base pairs. *Note*: The ADD test method was used.

SNP	Chromosome, BP, Allele	*p*-Value	Gene Symbol	Gene Name
rs2148655	13:42640069_T	8.32 × 10^−8^	*DGKH*	Diacylglycerol kinase
rs6448501	4:27188819_C	1.73 × 10^−7^	Intergenic (upstream of *LINC02261*)	Long Intergenic Non-Protein Coding RNA 2261
rs12508379	4:27172452_A	3.69 × 10^−7^	Intergenic (upstream of *LINC02261*)	Long Intergenic Non-Protein Coding RNA 2261
rs185335076	4:27163080_T	4.31 × 10^−7^	Intergenic (upstream of *LINC02261*)	Long Intergenic Non-Protein Coding RNA 2261
rs13132147	4:27166830_G	9.15 × 10^−7^	Intergenic (upstream of *LINC02261*)	Long Intergenic Non-Protein Coding RNA 2261
rs35956129	4:27166974_A	9.15 × 10^−7^	Intergenic (upstream of *LINC02261*)	Long Intergenic Non-Protein Coding RNA 2261
rs1275406	14:69592739_G	1.57 × 10^−6^	*DCAF5*	DDB1 And CUL4 Associated Factor 5
rs7684596	4:27215165_G	2.43 × 10^−6^	Intergenic (upstream of *LINC02261*)	Long Intergenic Non-Protein Coding RNA 2261
rs7684597	4:27215170_G	2.43 × 10^−6^	Intergenic (upstream of *LINC02261*)	Long Intergenic Non-Protein Coding RNA 2261
rs7684611	4:27215189_G	2.43 × 10^−6^	Intergenic (upstream of *LINC02261*)	Long Intergenic Non-Protein Coding RNA 2261

**Table 3:** Annotation of quantitative trait loci (eQTLs). *Abbreviations*: GTEx, The Genotype-Tissue Expression Project; GRCh38, Genome Reference Consortium Human Build 38. *Note*: The reference allele is mentioned first, followed by the alternative allele. GTEx-reported variants and chromosome coordinates are GRCh38 build-based.

SNP	eQTL Allele; Consequence	Gene Symbol	eQTL *p*-Value (Whole Blood)	Normalized Effect Size (NSE)
rs2477720	chr1_117062822_A_G_intron_variant	*TTF2*	2.80 × 10^−12^	−0.19
rs3746583	chr20_44936918_G_C_intron_variant	*PABPC1L*	5.90 × 10^−7^	−0.2
rs12402335	chr1_114593474_T_A_intron_variant	*DENND2C*	0.00065	0.00014
rs79095830	chr10_99787461_G_T_intron_variant	*ABCC2*	0.0059	0.057
rs1275415	chr14_69144993_C_T_intron_variant	*DCAF5*	0.013	−0.049
rs2001355	chr14_69094476_A_G_intron_variant	*DCAF5*	0.018	−0.047
rs1710989	chr14_69114519_C_A_intron_variant	*DCAF5*	0.018	−0.047
rs1892297	chr14_69121880_C_T_intron_variant	*DCAF5*	0.018	−0.047
rs1275407	chr14_69126735_G_A_intron_variant	*DCAF5*	0.018	−0.047
rs1147481	chr14_69131052_G_C_intron_variant	*DCAF5*	0.018	−0.047
rs1275414	chr14_69140079_G_A_intron_variant	*DCAF5*	0.018	−0.047
rs1105625	chr14_69082287_C_T_intron_variant	*DCAF5*	0.02	−0.046
rs10498527	chr14_69083045_C_T_intron_variant	*DCAF5*	0.02	−0.046
rs11158786	chr14_69086721_G_A_intron_variant	*DCAF5*	0.02	−0.046
rs11158789	chr14_69105286_A_C_intron_variant	*DCAF5*	0.02	−0.046
rs1275413	chr14_69128378_A_G_intron_variant	*DCAF5*	0.02	−0.046
rs11817729	chr10_59788419_G_A_downstream_gene_variant	*CCDC6*	0.028	−0.079
rs11158784	chr14_69086688_C_G_intron_variant	*DCAF5*	0.037	−0.043
rs11628126	chr14_69098476_T_G_intron_variant	*DCAF5*	0.037	−0.042
rs1710990	chr14_69108337_A_G_intron_variant	*DCAF5*	0.037	−0.043
rs9323521	chr14_69086410_A_G_intron_variant	*DCAF5*	0.039	−0.042
rs1275417	chr14_69150998_C_T _intron_variant	DCAF5	0.039	−0.042
rs11158785	chr14_69086703_T_C_intron_variant	*DCAF5*	0.039	−0.042
rs1626122	chr14_69100472_T_A_intron_variant	*DCAF5*	0.039	−0.042

## Data Availability

The data presented in this study are available on request from the corresponding author.

## Supplementary Materials

The following supporting information can be downloaded at: www.mdpi.com/article/10.3390/genes13050869/s1, Figure S1: GC corrected QQ-plot; Table S1: Population distribution of individuals genotyped using the two platforms i.e Immunochip and Global Screening Array (GSA); Table S2: NPY-LA ~ SNP associations for the top 348 SNPs; Table S3: Top molecules from the IPA pathway analysis of the 348 top annotated SNPs.

## References

[1] Burrack A.L., Martinov T., Fife B.T. (2017). T Cell-Mediated Beta Cell Destruction: Autoimmunity and Alloimmunity in the Context of Type 1 Diabetes. Front. Endocrinol..

[2] Pociot F., Kaur S., Nielsen L.B. (2016). Effects of the Genome on Immune Regulation in Type 1 Diabetes. Pediatric Diabetes.

[3] Robertson C.C., Inshaw J.R.J., Onengut-Gumuscu S., Chen W.-M., Santa Cruz D.F., Yang H., Cutler A.J., Crouch D.J.M., Farber E., Bridges S.L. (2021). Fine-Mapping, Trans-Ancestral and Genomic Analyses Identify Causal Variants, Cells, Genes and Drug Targets for Type 1 Diabetes. Nat. Genet..

[4] Clayton D.G. (2009). Prediction and Interaction in Complex Disease Genetics: Experience in Type 1 Diabetes. PLoS Genet..

[5] Rewers M., Hyöty H., Lernmark Å., Hagopian W., She J.-X., Schatz D., Ziegler A.-G., Toppari J., Akolkar B., The TEDDY Study Group (2018). The Environmental Determinants of Diabetes in the Young (TEDDY) Study: 2018 Update. Curr. Diabetes Rep..

[6] Lampasona V., Liberati D. (2016). Islet Autoantibodies. Curr. Diabetes Rep..

[7] Pociot F., Lernmark Å. (2016). Genetic Risk Factors for Type 1 Diabetes. Lancet.

[8] Törn C., Hadley D., Lee H.-S., Hagopian W., Lernmark Å., Simell O., Rewers M., Ziegler A., Schatz D., Akolkar B. (2015). Role of Type 1 Diabetes–Associated SNPs on Risk of Autoantibody Positivity in the TEDDY Study. Diabetes.

[9] Graham J., Hagopian W.A., Kockum I., Li L.S., Sanjeevi C.B., Lowe R.M., Schaefer J.B., Zarghami M., Day H.L., Landin-Olsson M. (2002). Genetic Effects on Age-Dependent Onset and Islet Cell Autoantibody Markers in Type 1 Diabetes. Diabetes.

[10] Brorsson C.A., Pociot F., The Type 1 Diabetes Genetics Consortium (2015). Shared Genetic Basis for Type 1 Diabetes, Islet Autoantibodies, and Autoantibodies Associated With Other Immune-Mediated Diseases in Families With Type 1 Diabetes. Diabetes Care.

[11] Regnell S.E., Lernmark Å. (2017). Early Prediction of Autoimmune (Type 1) Diabetes. Diabetologia.

[12] Brorsson C.A., Onengut S., Chen W.-M., Wenzlau J., Yu L., Baker P., Williams A.J.K., Bingley P.J., Hutton J.C., Eisenbarth G.S. (2015). Novel Association Between Immune-Mediated Susceptibility Loci and Persistent Autoantibody Positivity in Type 1 Diabetes. Diabetes.

[13] So M., Speake C., Steck A.K., Lundgren M., Colman P.G., Palmer J.P., Herold K.C., Greenbaum C.J. (2021). Advances in Type 1 Diabetes Prediction Using Islet Autoantibodies: Beyond a Simple Count. Endocr. Rev..

[14] Plagnol V., Howson J.M.M., Smyth D.J., Walker N., Hafler J.P., Wallace C., Stevens H., Jackson L., Simmonds M.J., Type 1 Diabetes Genetics Consortium (2011). Genome-Wide Association Analysis of Autoantibody Positivity in Type 1 Diabetes Cases. PLoS Genet..

[15] Hirai H., Miura J., Hu Y., Larsson H., Larsson K., Lernmark A., Ivarsson S.-A., Wu T., Kingman A., Tzioufas A.G. (2008). Selective Screening of Secretory Vesicle-Associated Proteins for Autoantigens in Type 1 Diabetes: VAMP2 and NPY Are New Minor Autoantigens. Clin. Immunol..

[16] Antonelli A., Tuomi T., Nannipieri M., Fallahi P., Nesti C., Okamoto H., Groop L., Ferrannini E. (2002). Autoimmunity to CD38 and GAD in Type I and Type II Diabetes: CD38 and HLA Genotypes and Clinical Phenotypes. Diabetologia.

[17] McLaughlin K.A., Tombs M.A., Christie M.R. (2020). Autoimmunity to tetraspanin-7 in type 1 diabetes. Med. Microbiol. Immunol..

[18] Tsuboi T., McMahon H.T., Rutter G.A. (2004). Mechanisms of Dense Core Vesicle Recapture Following “Kiss and Run” (“Cavicapture”) Exocytosis in Insulin-Secreting Cells. J. Biol. Chem..

[19] Skärstrand H., Vaziri-Sani F., Delli A.J., Törn C., Elding Larsson H., Ivarsson S., Agardh D., Lernmark Å., The Skåne Study group (2015). Neuropeptide Y Is a Minor Autoantigen in Newly Diagnosed Type 1 Diabetes Patients. Pediatr. Diabetes.

[20] Decressac M., Barker R.A. (2012). Neuropeptide Y and Its Role in CNS Disease and Repair. Exp. Neurol..

[21] Rodnoi P., Rajkumar M., Moin A.S.M., Georgia S.K., Butler A.E., Dhawan S. (2017). Neuropeptide Y Expression Marks Partially Differentiated β Cells in Mice and Humans. JCI Insight.

[22] Skärstrand H., Dahlin L.B., Lernmark Å., Vaziri-Sani F. (2013). Neuropeptide Y Autoantibodies in Patients with Long-Term Type 1 and Type 2 Diabetes and Neuropathy. J. Diabetes Complicat..

[23] Waeber G., Thompson N., Waeber B., Brunner H.R., Nicod P., Grouzmann E. (1993). Neuropeptide Y Expression and Regulation in a Differentiated Rat Insulin-Secreting Cell Line. Endocrinology.

[24] Cho Y.R., Kim C.W. (2004). Neuropeptide Y Promotes β-Cell Replication via Extracellular Signal-Regulated Kinase Activation. Biochem. Biophys. Res. Commun..

[25] Herzog H. (2012). 30Years of NPY Research. Neuropeptides.

[26] Mansachs S., Lind A., Lernmark A., Agardh D., Jensen A.K., Johannesen J., Pociot F. (2022). Neuropeptide Y Autoantibodies in Type 1 Diabetes: Associations to Islet Autoantibodies, Glycemic Control, β-Cell Function and Body Mass Index. Translational Type 1 Diabetes Research.

[27] Thorsen S.U., Pipper C.B., Mortensen H.B., Pociot F., Johannesen J., Svensson J. (2016). No Contribution of GAD-65 and IA-2 Autoantibodies around Time of Diagnosis to the Increasing Incidence of Juvenile Type 1 Diabetes: A 9-Year Nationwide Danish Study. Int. J. Endocrinol..

[28] Svensson J., Eising S., Mortensen H.B., Christiansen M., Laursen I., Lernmark Å., Nilsson A., Simonsen L.B., Carstensen B., Pociot F. (2012). High Levels of Immunoglobulin E and a Continuous Increase in Immunoglobulin G and Immunoglobulin M by Age in Children with Newly Diagnosed Type 1 Diabetes. Hum. Immunol..

[29] Madsen J.O.B., Herskin C.W., Zerahn B., Jensen A.K., Jørgensen N.R., Olsen B.S., Svensson J., Pociot F., Johannesen J. (2020). Bone Turnover Markers during the Remission Phase in Children and Adolescents with Type 1 Diabetes. Pediatr. Diabetes.

[30] Grubin C.E., Daniels T., Toivola B., Landin-Olsson M., Hagopian W.A., Li L., Karlsen A.E., Boel E., Michelsen B., Lernmark Å. (1994). A Novel Radioligand Binding Assay to Determine Diagnostic Accuracy of Isoform-Specific Glutamic Acid Decarboxylase Antibodies in Childhood IDDM. Diabetologia.

[31] Bingley P.J., Bonifacio E., Mueller P.W., Participating Laboratories (2003). Diabetes Antibody Standardization Program: First Assay Proficiency Evaluation. Diabetes.

[32] Bonifacio E., Yu L., Williams A.K., Eisenbarth G.S., Bingley P.J., Marcovina S.M., Adler K., Ziegler A.G., Mueller P.W., Schatz D.A. (2010). Harmonization of Glutamic Acid Decarboxylase and Islet Antigen-2 Autoantibody Assays for National Institute of Diabetes and Digestive and Kidney Diseases Consortia. J. Clin. Endocrinol. Metab..

[33] Mire-Sluis A.R., Gaines Das R., Lernmark Å. (2000). The World Health Organization International Collaborative Study for Islet Cell Antibodies. Diabetologia.

[34] Delaneau O., Marchini J., Zagury J.-F. (2012). A Linear Complexity Phasing Method for Thousands of Genomes. Nat. Methods.

[35] Howie B.N., Donnelly P., Marchini J. (2009). A Flexible and Accurate Genotype Imputation Method for the Next Generation of Genome-Wide Association Studies. PLoS Genet..

[36] Sudmant P.H., Rausch T., Gardner E.J., Handsaker R.E., Abyzov A., Huddleston J., Zhang Y., Ye K., Jun G., Hsi-Yang Fritz M. (2015). An Integrated Map of Structural Variation in 2,504 Human Genomes. Nature.

[37] Yasuda S., Kai M., Imai S., Takeishi K., Taketomi A., Toyota M., Kanoh H., Sakane F. (2009). Diacylglycerol Kinase η Augments C-Raf Activity and B-Raf/C-Raf Heterodimerization. J. Biol. Chem..

[38] Jin J., Arias E.E., Chen J., Harper J.W., Walter J.C. (2006). A Family of Diverse Cul4-Ddb1-Interacting Proteins Includes Cdt2, Which Is Required for S Phase Destruction of the Replication Factor Cdt1. Mol. Cell.

[39] Vujkovic M., Keaton J.M., Lynch J.A., Miller D.R., Zhou J., Tcheandjieu C., Huffman J.E., Assimes T.L., Lorenz K., Zhu X. (2020). Discovery of 318 New Risk Loci for Type 2 Diabetes and Related Vascular Outcomes among 1.4 Million Participants in a Multi-Ancestry Meta-Analysis. Nat. Genet..

[40] Mao X., He Z., Zhou F., Huang Y., Zhu G. (2019). Prognostic Significance and Molecular Mechanisms of Adenosine Triphosphate-Binding Cassette Subfamily C Members in Gastric Cancer. Medicine.

[41] Ben Saad A., Bruneau A., Mareux E., Lapalus M., Delaunay J.-L., Gonzales E., Jacquemin E., Aït-Slimane T., Falguières T. (2021). Molecular Regulation of Canalicular ABC Transporters. Int. J. Mol. Sci..

[42] Dinsmore C.J., Soriano P. (2018). MAPK and PI3K Signaling: At the Crossroads of Neural Crest Development. Dev. Biol..

[43] Kim E.K., Choi E.-J. (2010). Pathological Roles of MAPK Signaling Pathways in Human Diseases. Biochim. Biophys. Acta (BBA)-Mol. Basis Dis..

[44] Camaya I., Donnelly S., O’Brien B. (2022). Targeting the PI3K/Akt Signaling Pathway in Pancreatic Β-cells to Enhance Their Survival and Function: An Emerging Therapeutic Strategy for Type 1 Diabetes. J. Diabetes.

[45] Schultze S.M., Hemmings B.A., Niessen M., Tschopp O. (2012). PI3K/AKT, MAPK and AMPK Signalling: Protein Kinases in Glucose Homeostasis. Expert Rev. Mol. Med..

[46] Weston C.R., Davis R.J. (2007). The JNK Signal Transduction Pathway. Curr. Opin. Cell Biol..

[47] Maffei A., Lembo G., Carnevale D. (2018). PI3Kinases in Diabetes Mellitus and Its Related Complications. Int. J. Mol. Sci..

[48] Krebs D.L., Chehal M.K., Sio A., Huntington N.D., Da M.L., Ziltener P., Inglese M., Kountouri N., Priatel J.J., Jones J. (2012). Lyn-Dependent Signaling Regulates the Innate Immune Response by Controlling Dendritic Cell Activation of NK Cells. J. Immunol..

[49] Hurst J.H., Dohlman H.G. (2013). Dynamic Ubiquitination of the Mitogen-Activated Protein Kinase Kinase (MAPKK) Ste7 Determines Mitogen-Activated Protein Kinase (MAPK) Specificity. J. Biol. Chem..

[50] Weber A., Wasiliew P., Kracht M. (2010). Interleukin-1 (IL-1) Pathway. Sci. Signal..

[51] Pickart C.M., Fushman D. (2004). Polyubiquitin Chains: Polymeric Protein Signals. Curr. Opin. Chem. Biol..

[52] Sun W., Zhu P., Shi Y., Zhang C., Huang X., Liang S., Song Z., Lin S. (2017). Current Views on Neuropeptide Y and Diabetes-Related Atherosclerosis. Diabetes Vasc. Dis. Res..

[53] Kim Y.J., Bi S. (2016). Knockdown of Neuropeptide Y in the Dorsomedial Hypothalamus Reverses High-Fat Diet-Induced Obesity and Impaired Glucose Tolerance in Rats. Am. J. Physiol.-Regul. Integr. Comp. Physiol..

[54] Bi S., Li L. (2013). Browning of White Adipose Tissue: Role of Hypothalamic Signaling: Central Role in Browning of White Adipose Tissue. Ann. N. Y. Acad. Sci..

[55] Machida Y., Bruinsma C., Hallinger D.R., Roper S.M., Garcia E., Trevino M.B., Nadler J., Ahima R., Imai Y. (2014). Pancreatic Islet Neuropeptide Y Overexpression Has Minimal Effect on Islet Morphology and β-Cell Adaptation to High-Fat Diet. Endocrinology.

[56] Garcia F.D., Coquerel Q., do Rego J.-C., Cravezic A., Bole-Feysot C., Kiive E., Déchelotte P., Harro J., Fetissov S.O. (2012). Anti-Neuropeptide Y Plasma Immunoglobulins in Relation to Mood and Appetite in Depressive Disorder. Psychoneuroendocrinology.

[57] Hao F., Pysz M.A., Curry K.J., Haas K.N., Seedhouse S.J., Black A.R., Black J.D. (2011). Protein Kinase Cα Signaling Regulates Inhibitor of DNA Binding 1 in the Intestinal Epithelium. J. Biol. Chem..

[58] GeneCards—The Human Gene Database. www.genecards.org.

[59] Pellatt G.C. (2008). Neurogenic Continence. Part 1: Pathophysiology and Quality of Life. Br. J. Nurs..

[60] Bolinder J., Sjöberg S., Persson A., Ahrén B., Sundkvist G. (2002). Autonomic Neuropathy Is Associated with Impaired Pancreatic Polypeptide and Neuropeptide Y Responses to Insulin-Induced Hypoglycaemia in Type I Diabetic Patients. Diabetologia.

[61] Mitoma H., Manto M., Hampe C.S. (2017). Pathogenic Roles of Glutamic Acid Decarboxylase 65 Autoantibodies in Cerebellar Ataxias. J. Immunol. Res..

[62] Manchia M., Squassina A., Congiu D., Chillotti C., Ardau R., Severino G., Del Zompo M. (2009). Interacting Genes in Lithium Prophylaxis: Preliminary Results of an Exploratory Analysis on the Role of DGKH and NR1D1 Gene Polymorphisms in 199 Sardinian Bipolar Patients. Neurosci. Lett..

[63] Ota T., Suzuki Y., Nishikawa T., Otsuki T., Sugiyama T., Irie R., Wakamatsu A., Hayashi K., Sato H., Nagai K. (2004). Complete Sequencing and Characterization of 21,243 Full-Length Human CDNAs. Nat. Genet..

[64] Hashimoto R., Ikeda M., Yamashita F., Ohi K., Yamamori H., Yasuda Y., Fujimoto M., Fukunaga M., Nemoto K., Takahashi T. (2014). Common Variants at 1p36 Are Associated with Superior Frontal Gyrus Volume. Transl. Psychiatry.

[65] Wang Y., Gan H., Su F., Zhang H., Wang S., Xian J. (2016). Role of MAPK/ERK Signal Pathway in Recurrent Miscarriage Patients by Case-Control Analysis. Int. J. Clin. Exp. Pathol..

[66] Jarosz-Popek J., Wolska M., Gasecka A., Czajka P., Jakubik D., Sharif L., Adem T., Liu W.-L., Mirowska-Guzel D., Postula M. (2020). The Importance of Non-Coding RNAs in Neurodegenerative Processes of Diabetes-Related Molecular Pathways. J. Clin. Med..

[67] Schlossmann J., Ammendola A., Ashman K., Zong X., Huber A., Neubauer G., Wang G.-X., Allescher H.-D., Korth M., Wilm M. (2000). Regulation of Intracellular Calcium by a Signalling Complex of IRAG, IP3 Receptor and CGMP Kinase Iβ. Nature.

[68] McCullough L.A., Egan T.M., Westfall T.C. (1998). Neuropeptide Y Receptors Involved in Calcium Channel Regulation in PC12 Cells. Regul. Pept..

[69] Hoefen R.J., Berk B.C. (2002). The Role of MAP Kinases in Endothelial Activation. Vasc. Pharmacol..

[70] Graf G.A., Cohen J.C., Hobbs H.H. (2004). Missense Mutations in ABCG5 and ABCG8 Disrupt Heterodimerization and Trafficking. J. Biol. Chem..

[71] Kakko T., Jaakkola U., Raitakari O.T., Kallio J. (2011). Inflammatory Effects of Blood Leukocytes: Association with Vascular Function in Neuropeptide Y Proline 7-Genotyped Type 2 Diabetes Patients. Diabetes Vasc. Dis. Res..

