# Supplementary material for: Genetic Variants Associated with Neuropeptide Y Autoantibody Levels in Newly Diagnosed Individuals with Type 1 Diabetes
Source: Genes (Basel). 2022 May 12;13(5):869. doi: 10.3390/genes13050869 (PMC9142038; doi:10.3390/genes13050869)
Supplement: Supplementary file 1 [file genes-13-00869-s001.zip › Supplementary Materials.pdf]

**Supplementary Materials:**

**Table S1.** Population distribution of individuals genotyped using the two platforms i.e Immunochip and Global Screening Array (GSA).

| Population | Immunochip | GSA |
|---|---|---|
| **Danish** | 468 | 68 |
| **European/Turkish** | 11 | 2 |
| **Other** | 7 | 4 |
| **Total** | 479 | 70 |

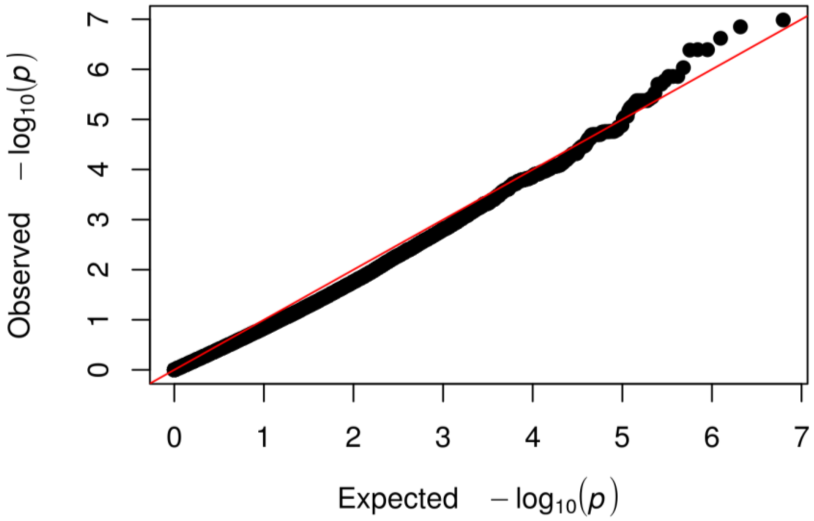


**Figure S1:** GC corrected QQ-plot.

**Table S3**: Top molecules from the IPA pathway analysis of the 348 top annotated SNPs. Genes in bold correspond to genes with SNPs mapping to eQTL or in association with NPY-LA. *Abbreviations*: IPA, Ingenuity Pathway Analysis; SNPs, Single nucleotide polymorphisms; NPY-LA, Neuropeptide Y autoantibodies; FXR, nuclear receptor Farnesoid X Receptor; RXR, retinoid X receptor; eQTL, quantitative trait loci.

| Gene Symbol | Gene Name | SNP | Bonferroni | Molecular and biological function |
|---|---|---|---|---|
| *DGKH* | diacylglycerol kinase eta | rs2148655 | $8.32 \times 10^{-8}$ | Diacylglycerol kinase (DGK) enzyme family regulates intracellular concentrations of phosphatidic acid and diacylglycerol, promoting cell growth by activating the Ras/B-Raf/C-Raf/MEK/ERK signaling pathway. *DGKH* is a protein-coding gene, nucleotide binding, enables NAD+ kinase activity, and enables ATP binding. |
| *DCAF5* | DDB1 and CUL4 associated factor 5 | rs1275406 | $1.57 \times 10^{-6}$ | Substrate receptor for CUL4-DDB1 E3 ubiquitin-protein ligase complex. *DCAF5* affects post-translational protein modification and is a protein-coding gene. |

| | | | | |
|---|---|---|---|---|
| *LRP1B* | LDL receptor related protein 1B | rs6755822 | 5.05 ×10$^{-6}$ | Enables low-density lipoprotein particle receptor activity. |
| *SND1* | staphylococcal nuclease and tudor domain containing 1 | rs3757779 | 7.99 ×10$^{-6}$ | Endonuclease that mediates miRNA decay of both protein-free and AGO2-loaded miRNAs, bridging factor between STAT6 and the basal transcription factor. |
| *LOC105373781* | uncharacterized | rs1483242 | 8.88 ×10$^{-6}$ | Affiliated with the ncRNA class. |
| *FAM230E (includes others)* | family with sequence similarity 230 member | rs375428700 | 1.24 ×10$^{-5}$ | Affiliated with the lncRNA class. |
| *LOC105373720* | uncharacterized | rs1483242 | 8.88 ×10$^{-6}$ | |
| *TBR1* | T-box brain transcription factor 1 | rs890076 | 1.62 ×10$^{-5}$ | A member of a conserved family of genes that share the common T-box DNA-binding domain. T-box genes encode transcription factors involved in the regulation of numerous developmental processes. |
| *CAPZA1* | capping actin protein of muscle Z-line subunit alpha 1 | rs12566829 | 1.66 ×10$^{-5}$ | Actin cytoskeleton organization and endoplasmic reticulum to Golgi vesicle-mediated transport. |
| ***PABPC1L*** | poly(A) binding protein cytoplasmic 1 like | rs3746583 | 1.96 ×10$^{-5}$ | Binds to the polyA tails of mRNAs to regulate mRNA stability and translation. |
| ***ABCC2*** | ATP binding cassette subfamily C member 2 | rs79095830 | 2.02 ×10$^{-5}$ | ATP-dependent transporter of the ATP-binding cassette (ABC) family that binds and hydrolyzes ATP to enable active transport of various substrates including many drugs, toxicants, and endogenous compounds across cell membranes. |
